# Trends and patterns of the double burden of malnutrition (DBM) in Peru: a pooled analysis of 129,159 mother–child dyads

**DOI:** 10.1038/s41366-020-00725-x

**Published:** 2021-01-05

**Authors:** Marco Pomati, Daniel Mendoza-Quispe, Cecilia Anza-Ramirez, Akram Hernández-Vásquez, Rodrigo M. Carrillo Larco, Gabriela Fernandez, Shailen Nandy, J. Jaime Miranda, Antonio Bernabé-Ortiz

**Affiliations:** 1 School of Social Sciences, Cardiff University, Glamorgan Building, King Edward VII Avenue, Cardiff, Wales CF24 3PG UK; 2 CRONICAS Center of Excellence in Chronic Diseases, Universidad Peruana Cayetano Heredia, Lima, Peru; 3 Department of Epidemiology and Biostatistics, School of Public Health, Imperial College London, London, UK; 4 Federal University of Rio de Janeiro, Rio de Janeiro, Brazil; 5 School of Medicine, Universidad Peruana Cayetano Heredia, Lima, Peru

**Keywords:** Malnutrition, Epidemiology

## Abstract

**Background:**

This study aims to evaluate trends of DBM in Peru over the last 20 years.

**Methods:**

Using individual-level data collected in nationally representative household surveys from Peru between 1996 and 2017, we analysed trends in the prevalence and patterning of the DBM. We classified the nutritional status of children and their mothers as undernourished (either underweight, stunted or wasted for children), normal, overweight or obese. Children classified as experiencing the DBM were those undernourished and living with an overweight or obese mother. We also fitted logistic regression models to evaluate the probability of children having an overweight/obese mother across subgroups of socioeconomic status, place of residence and education.

**Results:**

The overall percentage of children experiencing the DBM in 2016 was 7%, and constitutes ~203,600 children (90% of whom were stunted). Between 1996 and 2016, undernourished children have seen the largest relative increase in the risk of having an overweight mother (31% vs. 37%) or obese mother (6% vs. 17%); however, due to the substantial decrease in the absolute number of undernourished children, the DBM has not grown. Moreover, all children, irrespective of their own nutritional status, are now more likely to live with an overweight or obese mother, a consistent pattern across wealth, location and education subgroups, and all regions of Peru.

**Conclusions:**

DBM prevalence in Peru has decreased, although the number of DBM cases is estimated to be above 200,000. In addition, all children are now more likely to live with overweight or obese mothers. The basic pattern has shifted from one of undernourished children whose mothers have a ‘normal’ BMI, to one where now most children have a ‘normal’ or healthy anthropometric status, but whose mothers are overweight or obese. This suggest that Peru is on the cusp of a major public health challenge requiring significant action.

## Introduction

Peru, along with many other low- and middle-income countries (LMICs), has undergone a dramatic nutritional transformation in the past 30 years, with obesity rates increasing among women in the face of declining and sometimes persistent child undernutrition [1, 2]. This shift has evolved into the so-called double burden of malnutrition (DBM), where undernutrition and overnutrition coexist within individuals, households, and at the population level [3, 4]. Globally, the DBM prevalence at household level is generally below 10%, but its prevalence in LMICs is increasing, particularly in Latin America [5].

As part of wider social and economic developments, the nutrition transition in LMICs started later but evolved faster—decades rather than centuries; these rapid changes provided fewer opportunities to accommodate dietary and social changes, with a subsequent negative impact on health [6]. Understanding the DBM, both in children and adults, is important as nutritional development affects people’s learning and cognitive processes, their physical response to infectious disease, and the development of chronic conditions in later life [7–9]. In addition to health outcomes, both extremes of the nutrition spectrum (undernutrition and overnutrition) affect productivity and thus economics at the individual, household, and health system levels. Despite this, epidemiological evidence has classically focused on addressing either undernutrition or obesity rather than considering both jointly.

A growing body of research demonstrates that the DBM is a particular challenge for certain social groups, including the urban poor, the rural rich and people living in slum conditions [10–13].

Peru is experiencing a rapid nutrition transition, where the patterning and dynamics of the DBM are not yet fully understood. Obesity prevalence among Peruvian women more than doubled from 9% in the mid-1990s, to almost 20% by 2011 [14]; during the same period, child undernutrition—assessed by either stunting, wasting or underweight—declined significantly [15]. These dynamics place Peru in the third/fourth phase of the nutrition transition, meaning it is at high risk of developing a significant DBM [16]. Although the estimated national prevalence of DBM in mother–child dyads in Peru has been at 8% from 1991 to 2004 [17], these data may hide significant within-country variations. For example, stunting (i.e. low height-for-age) rates are not uniform across the country, ranging from 13% on the coast to 44% in more highland areas; other forms of malnutrition (e.g. wasting) also vary widely within Peru [16]. Identifying these vulnerable clusters, in terms of location and determinants, would enable the focusing of (scarce) resources where they are most needed to have the greatest impact.

This study aims to understand and model trends and patterns of DBM in mother–child dyads from Peru over the last 20 years. Our findings for Peru will contribute to the growing international literature on the DBM, adding potential options for addressing it in LMICs.

## Methods

### Study design

We conducted a cross-sectional, population-based, secondary analysis of the Peruvian Demographic and Health Survey (DHS). This survey is run by the National Institute of Statistics and Informatics (INEI in Spanish) and provides representative information at the region and country level, comprising a reliable source of data for understanding nutritional profiles across Peru in the last twenty years. We used data for the years 1996, 2000, 2004/2008, 2009/2011 and 2015/2017 to analyse DBM trends and focused on the 2015/17 data to look at more detailed patterning. Datasets were obtained from the DHS (https://dhsprogram.com/) or INEI (https://proyectos.inei.gob.pe/endes/) websites. Descriptions of the sampling and other methodological procedures for the survey for each round/year are available at the INEI website [18].

### Population sample and sampling

All DHS surveys used stratified two-stage probability samples. The unit of analysis was the child. Clusters and households were the primary and secondary sampling units, respectively. INEI/DHS reports showed the samples matched known population characteristics. Weights provided by INEI/DHS were post-stratified to adjust for non-response which was generally very low (household response rates were 98% or higher across the five analysed samples) [19–23]. Sociodemographic and anthropometric data from children under 5 and their mothers (aged 15–49 years) were retrieved for selected survey years. Although we were only able to analyse data on children aged 0–5 with complete information on their mother’s and their own heights and weights, the main socio-economic characteristics did not vary significantly between the whole sample and the analysed sample (we compared the percentage of children in rural areas, the percentage who are male, who have a mother with secondary and higher qualification and who live in poor areas).

### Definition of variables

#### Outcome variable

Our primary outcome was DBM, defined at child level as the coexistence of an undernourished child and an overweight/obese mother [24–27]. Data on the DBM were also decomposed into the two components of this definition, child undernutrition and maternal overweight/obesity, to highlight the different combinations and permutations of maternal and child nutritional status. We did not consider instances where the mothers were underweight and children overweight or obese because of small sample size issues due to the low prevalence of maternal underweight (<1%). Children’s nutritional status was assessed using the Composite Index of Anthropometric Failure [12], with children who experienced either stunting, wasting, or underweight classified as being ‘undernourished’. Stunting in children was identified if their height-for-age *Z*-scores were <−2 standard deviations (SD) from the 2006 WHO international reference population [28]; children with wasting were those whose weight-for-height *Z*-scores were <−2 SD; and underweight children were those whose weight-for-age Z scores were <−2 SD. Children with high weight-for-height *Z*-scores were classed as overweight (>2 SD) or obese (>3 SD). These 2006 WHO criteria are based on the analysis of growth trajectories of adequately-nourished children from Ghana, India, Norway, Brazil, Oman, and North America and were created to be widely comparable across a wide range of socio-economic contexts [29, 30]. Maternal nutritional status was defined using body mass index (BMI), calculated by dividing weight in kilograms by the square of height in metres. Categories were underweight for BMI of <18.5 kg/m^2^ [2], normal if BMI > 18.5 kg/m^2^ and <25 kg/m^2^, overweight if BMI ≥ 25 kg/m^2^ and <30 kg/m^2^, and obese if BMI ≥ 30 kg/m^2^. Children and mothers with missing or anthropometric measurements considered invalid (<−5SD or >+5 SD) were excluded from analyses.

#### Covariates of interest

*Household-level variables* included place of residence (region, and urban vs. rural), socioeconomic status (derived from the asset-based wealth index, split in tertiles—poor vs. middle vs. rich), and year of survey. *Mother-level variables* included age in years and highest educational attainment (no education vs. primary vs. secondary vs. higher). *Child-level variables* included sex and age in months.

### Statistical analysis

All analyses were carried out at the child level in the program R, using post-stratified sample weights. (see details above). Standard errors were corrected for lack of error independence due to child cases sharing the same mother (15%) using complex sampling procedures. The tables presented in this paper report either the percentage or total number of children under 5 years old in Peru for the respective year. The total number of children in any table was obtained by multiplying the estimated percentage (using post-stratification weights) by the annual number of children in Peru, derived using the United Nations World Population Prospects data (version 2019), specifically the total population (both sexes combined) by 5-year age group. Because for each child we also consider their mother’s information, we refer to our cases as dyads (i.e. pairs). Two children with the same mother constitute two separate cases (i.e. two separate dyads). First, we described trends in the prevalence of the DBM in mother–child dyads in the selected years by providing survey estimates for each year. Thereafter, the DBM and its components were analysed in separate logistic regression models using the pooled data. Logistic regression (also known as logit regression), allows us to model binary dependent variables as a function of a set of independent variables. In this paper we fit a logistic regression model to analyse the probability of children having overweight/obese mothers across subgroups of socioeconomic status, place of residence and highest mother educational attainment. We present regression coefficients for every independent variable in the form of odds ratios (OR). These show whether each variable increases (OR > 1) or decreases (OR < 1) the odds of having an overweight/obese mother, controlling for all other independent variables. Multicollinearity between the independent variables was generally low (all VIF < 2) [31]. Pooling datasets with adjacent years (i.e. 2004/2008, 2009/2011 and 2015/2017) allows us to estimate percentages for small groups (e.g. specific types of child undernourishment by maternal weight status) with a smaller margin of error. Logistic regression models were adjusted by covariates of interest and year of the survey, and adjusted predicted probabilities were computed from these models.

### Ethics

Participants in the Peruvian DHS provided consent and standard ethical issues concerned with maintaining respondent confidentiality were addressed. This study was a secondary analysis of anonymous, publicly available data. The protocol of this study (code 102441) was approved by the Institutional Ethics Committee of the *Universidad Peruana Cayetano Heredia* (Lima, Peru).

## Results

### Population description

Data from 129,159 children (93% of the overall DHS sample of children under 5; range between 88% and 97%) with valid personal and maternal nutritional information was analysed (see Table 1).

**Table 1:** Sample description by Demographic Health Survey year.

Sample	1996	2000	2008	2010	2016	Total
Eligible children under 5	16,600	13,130	11,423	27,727	69,613	138,493
Children with valid personal nutritional data	14,997	11,671	11,671	26,914	67,698	132,951
Children with valid personal and maternal nutritional data	14,812	11,565	10,384	26,805	65,593	129,159
Percentage of eligible children under 5 with valid personal and maternal nutritional data	89%	88%	91%	97%	94%	93%

There were roughly 2.8 million children under the age of 5 in Peru in 2016. Of these, the large majority (78%) were neither underweight nor overweight (“normal” status), whereas 22% were either undernourished, overweight or obese. Specifically, around 14% of children under 5 were undernourished (i.e. wasting, stunting or underweight), and 8% were either overweight or obese. This is part of an overall historical trajectory; with the number and percentage of undernourished children under 5 in Peru falling between 1996 and 2016 (Table 2). In contrast, in 2016 around 40% of the children had overweight mothers, 22% had obese mothers, and only 1% had underweight mothers. Data on pregnant mothers are shown separately as the reliability of BMI as an indicator of nutritional status during pregnancy is problematic [32].

**Table 2:** Estimated nutritional status of children aged 0–5 years and mothers (number and percentage of children) living in Peru between 1996 and 2016.

	1996	2000	2008	2010	2016
Child status					
°Undernourished	30% (934,449)	29% (864,892)	27% (798,623)	22% (665,757)	14% (397,904)
°Normal	60% (1,865,464)	59% (1,762,700)	63% (1,870,682)	68% (2,056,928)	78% (2,178,349)
°Overweight	8% (254,661)	9% (276,799)	8% (236,752)	8% (227,927)	6% (178,421)
°Obese	2% (70,219)	3% (95,529)	2% (64,425)	2% (53,840)	2% (45,200)
Mother status					
°Underweight	1% (32,872)	1% (19,516)	1% (25,770)	1% (27,033)	1% (27,151)
°Normal	51% (1,580,993)	50% (1,490,436)	46% (1,360,937)	42% (1,265,118)	34% (957,188)
°Overweight	33% (1,039,190)	33% (997,621)	36% (1,055,878)	37% (1,112,344)	39% (1,097,277)
°Obese	8% (264,633)	11% (315,468)	12% (365,740)	15% (458,553)	22% (619,871)
°Pregnant	7% (207,104)	6% (176,879)	5% (162,156)	4% (131,403)	4% (98,386)

Authors’ calculations using Peru DHS

### DBM composition and trends over time

The most consistent finding is the large proportion of children living with an overweight or obese mother, irrespective of the child’s own nutritional status (Table 3). Regarding DBM in 2016, undernourished children were less likely to have an overweight mother than children in other groups (i.e. 35% compared to roughly 40% of normal, overweight and obese children). Undernourished children were also less likely to have an obese mother, compared to children in other groups. By collapsing the percentage of overweight and obese mothers we observed that 51% (35% + 16%) of undernourished child had an overweight mother, compared to 62% (40% + 22%) for normal children, 74% (42% + 32%) for overweight children and 80% (39% + 41%) for obese children. Overall, the percentage of children experiencing the DBM was 7%, and constitutes approximately 203,600 children. Among undernourished children, those having mothers with ‘normal’ BMI are 43%, and constitute ~170,000 children, or around 6% of all children under 5 years in 2016.

**Table 3:** Child–mother nutritional status composition in 2016.

Child	Mother	%	%of all children	*N*
**Undernourished**	Underweight	1	0	4895
	Normal	43	6	169,761
	**Overweight**	**35**	**5**	**139,486**
	**Obese**	**16**	**2**	**64,147**
	Pregnant	5	1	19,405
Normal	Underweight	1	1	21,269
	Normal	34	26	738,025
	Overweight	40	31	865,643
	Obese	22	17	480,423
	Pregnant	3	3	73,081
Overweight	Underweight	1	0	941
	Normal	23	1	41,700
	Overweight	42	3	74,592
	Obese	32	2	56,989
	Pregnant	3	0	4601
Obese	Underweight	0	0	47
	Normal	17	0	7702
	Overweight	39	1	17,556
	Obese	41	1	18,311
	Pregnant	3	0	1298

Authors’ calculations using Peru’s DHS (2015, 2016, 2017)

Bold categories shown estimations of the double burden of malnutrition (i.e. 7% of the total)

Between 1996 and 2016 (Table 4), undernourished children have seen the largest relative (rather than absolute) increase in the risk of having an overweight mother (31% in 1996 vs. 37% in 2016) and the risk of having an obese mother (6% in 1996 vs. 17% in 2016), on average a fifth and a third more likely, accordingly. However, children who are not undernourished (i.e. who are either ‘normal’, overweight or obese) have on average seen much larger absolute (rather than relative) increases in the risk of having an overweight or obese mother; thus, these dyads were and are substantially more likely to occur overall (as shown in Table 3).

**Table 4:** Trends in maternal nutritional status according to nutritional status of the child.

Child	Mother	1996	2000	2008	2010	2016	Relative change 1996–2016 (%)
Undernourished	Normal	62%	61%	58%	55%	45%	−27
	Overweight	31%	30%	32%	34%	37%	+19
	Obese	6%	8%	9%	11%	17%	+183
Normal	Normal	52%	52%	46%	42%	35%	−33
	Overweight	36%	37%	40%	40%	41%	+14
	Obese	10%	11%	13%	16%	23%	+130
Overweight	Normal	41%	36%	39%	32%	24%	−41
	Overweight	46%	43%	40%	40%	43%	−7
	Obese	13%	21%	21%	27%	33%	+154
Obese	Normal	47%	49%	37%	27%	18%	−62
	Overweight	36%	39%	43%	45%	40%	+11
	Obese	17%	12%	20%	27%	42%	+147

Authors’ calculations using Peru’s DHS. Pregnant mothers were excluded.

The disaggregated composition of the DBM in Table 5 presents the patterning of combinations of child and maternal nutritional status; it shows that stunting is the main manifestation of undernourishment in children, and this pattern has remained constant over time. Thus, almost all children (over 90%) who contribute to the DBM in Peru were stunted—i.e. exhibiting long-term, chronic malnutrition, either on its own or in combination with other forms of malnutrition (i.e. groups IV—stunted and underweight and V—stunted only). The total number and the percentage of children who suffer from DBM are presented in the bottom two rows. Both the number and the percentage of undernourished children has decreased between 1996 (313,000 children, 10% of all children in 1996) and 2016 (203,000 or 7% of all children in 2016).

**Table 5:** Decomposition of the DBM in Peru over time.

Child Status//Mother Status	1996	2000	2008	2010	2016
I: Child: Wasted only//Mother: Overweight/Obese	3% (8802)	2% (5294)	2% (5838)	1% (2044)	1% (2197)
II: Child: Wasted & underweight//Mother: Overweight/Obese	1% (3046)	1% (3499)	1% (2991)	1% (1937)	2% (4244)
III: Child: Wasted, stunted & underweight/Mother: Overweight/Obese	1% (2278)	1% (1884)	0% (896)	1% (2278)	1% (2652)
IV: Child: Stunted & Underweight//Mother: Overweight/Obese	10% (32,832)	10% (31,597)	12% (35,182)	15% (40,818)	15% (29,688)
V: Child: Stunted only//Mother: Overweight/Obese	85% (264,851)	85% (259,566)	85% (255,799)	82% (226,560)	79% (160,429)
VI: Child: Underweight only//Mother: Overweight/Obese	0% (1327)	1% (1959)	0% (1296)	1% (2232)	2% (4424)
Total DBM	313,136	303,800	302,000	275,868	203,633
Percentage of all children	10%	10%	10%	9%	7%

Authors’ calculations using Peru’s DHS. Percentage of children experiencing any Anthropometric Failure and with overweight or obese mothers by year. Children with pregnant mothers were not considered DBM.

### DBM within socioeconomic groups

Although there were socio-economic variations, on the whole, a substantial increase in the percentage of ‘normal’ children with obese mothers were consistent across all regions of Peru (see Figure A1 in Online Supplement) as well as across wealth quintiles and rural and urban areas (Figure A2) and levels of education (Figure A3).

**Fig. 1:**
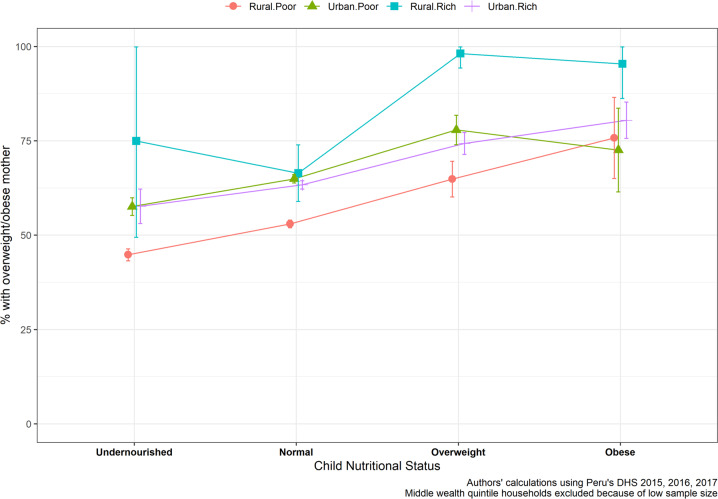
Relationship between geography, wealth and maternal obesity. Percentage of children in 2016 with an overweight/obese mother by household wealth index status and rurality (2015–17 data). Sample estimates of percentage of children with overweight/obese mother according to rural/urban and wealth index household status. Rural poor = pink circles. Urban Poor = green triangle. Rural Rich = blue square. Urban rich = purple line.

We also find evidence that the association between child and maternal nutritional status in 2016 presented in Table 4 remains broadly similarly across wealth quintiles (see Fig. 1). For example, among urban households in 2016, the chances of a child having an overweight or obese mother are greater for overweight and obese children, and this pattern was similar across wealth quantiles (Fig. 1). The exception to the latter pattern is among rural children, where the chances of having an overweight or obese mother are disproportionately greater among overweight children in rural rich households. Accordingly, virtually all young children from rural rich households who were overweight or obese also had overweight or obese mothers. The logistic regression model confirms that overweight or obese children living in rich rural households are the most likely to be living with an overweight/obese mother. In fact, the predicted probabilities for this model (see Table A1 and Figure A4 in Online Supplement) match very closely the percentages in Figure A3, despite adjusting for age of child and mother as well as mother’s level of education. Our model also suggests that a higher (above secondary) maternal level of education is generally associated with slightly lower odds (OR = 0.62, i.e. 38% lower, *p* < 0.05) of overweight or obesity in mothers compared to households with primary or secondary levels of education (even when controlling for the socioeconomic status of the household). The risk of DBM for different age groups is relatively similar. It is 8% for those 0–2 years and 11% for those who are older. Our regression models also suggest that once we control for other characteristics these age differences are not statistically significant.

## Discussion

Over the last three decades, the overall percentage of children who are undernourished and whose mothers are either overweight or obese (the classic notion of the DBM) has decreased over time from about 10% in 1996 to 7% in 2016. In this period (1996–2016), Peru has achieved an impressive reduction in child malnutrition; this manifests today with a substantial proportion of the child population having a ‘normal’ (or healthy) anthropometric profile. However, this change has occurred alongside a steady and significant increase in children having overweight or obese mothers, a change consistent across a range of socioeconomic variables (i.e. household wealth quintiles and residence in rural and urban areas). In addition, the basic pattern of mother–child dyad has shifted from one of undernourished children with mothers who had a ‘normal’ BMI, to one where now most children are classed as having a ‘normal’ or healthy anthropometric status, but whose mothers are now either overweight or obese.

With regards the DBM, the risk for an undernourished child of having an obese mother has increased, but this is also the case for all other children (i.e. be they normal or overweight). Moreover, there has been a substantial decrease in the absolute number of undernourished children meaning that on average, the number of undernourished children with overweight or obese parents has generally declined since 1996. We also find little evidence that the composition of the DBM has changed. Over 90% of children classed in ‘double burden’ households are stunted and this has changed little since the mid-1990s, suggesting there was no sizeable change in the percentage of children with an obese mother experiencing a combination of wasting and stunting simultaneously. This reiterates the finding that the driver of the DBM in Peru is increasingly one of over-nutrition (or excessive intake of bad nutrients) rather than undernutrition. We find little evidence of regional variation in these drivers and trajectories, but it is quite possible that contextual effects are present at a much more local level; more detailed, spatial analysis which takes into account the distribution of health facilities and the availability of certain types of food could form the basis for future research, as would qualitative studies which investigate the rise of obesity due to growing consumption of ultra-processed foods [33], and the influence of marketing on household food purchasing decisions [34].

### Relevance for public health

In any country, socioeconomic disparities, poverty, food insecurity, non-healthy lifestyles and other factors all reduce the ability to maintain metabolic homoeostasis, thus elevating the overall risk of the DBM and other non-communicable diseases [6]. The window of opportunity to act during a child’s first 1000 days of life warrants special focus and interest in mother–child dyads [4]. Intergenerational transmission of malnutrition through lower maternal educational levels, inadequate household infrastructure, lack of breastfeeding and consumption of poor-quality diets and non-healthy lifestyle factors are avenues through which interventions to reduce the DBM could be considered and addressed [4, 35].

Peru, like most LMICs, is undergoing sizeable social and economic developments that are resulting in rapid health and nutrition transitions; these dynamics mean countries are moving from contexts where the primary health challenges have been communicable diseases and undernutrition to ones where these persist, but new ones—such as the rise of chronic non-communicable disease and the diseases of overnutrition—have arisen, both which combine to place a strain and demand on underdeveloped health systems.

Children in rural areas are still at greater risk of experiencing the DBM. Moreover, progress in narrowing the gap between poorer and richer has been slower than in urban areas. Indeed, poor children in rural areas are still at greater risk of being undernourished, but are also experiencing an increase in maternal overweight. Educational attainment in these areas is still behind urban areas, which has been associated with lower risk of maternal overweight.

The fight against all forms of malnutrition is ingrained in the Sustainable Development Goals, which explicitly call for reductions in child wasting and stunting [36], and also greater efforts to tackle non-communicable diseases, like those associated with over-nutrition (like diabetes and heart disease). Peru made real progress in reducing child undernutrition, nearly halving it between 1996 and 2016. The share of overweight and obese children has remained relatively low and stable (at between 8–10%), which is comparable to other countries in the region. However, overweight is growing and already presenting significant public health challenges in adults and will do too for children growing into overweight/obese young adults. These changes are in line with increases in obesity globally, as are increases in the concomitant challenges—diabetes has increased and global targets, for example are unlikely to be met [37]. We find some preliminary evidence that higher levels of education may result in lower odds of maternal overweight but because the increase in maternal overweight in Peru has also occurred among mothers with higher levels of education, it seems unlikely that focusing solely on education will reverse or stall this overall trend.

The implications of a global growth in the DBM are now widely acknowledged. The challenges placed on health services and population health are non-trivial, particularly given the pace of change and the complex nature of the socio-economic drivers of the nutritional transition typified by the role of personal choice, the influence of food marketing, the nutritional contribution of unhealthy, ultra-processed but socially-desirable fast, ‘modern’ and convenient foods, all wrapped up by growing social inequality and modern, time-poor lifestyles. The greater abundance, affordability and apparent desirability of ‘modern’, processed foods has boosted demand among populations in LMICs, many of whom until relatively recently lacked access to them. Demand is stimulated and built by pervasive and persistent marketing campaigns, and the effective normalisation of foods high in fat, sugar and calories but low in substantive nutritional value. The outcomes of this are manifested in changing body shapes (i.e. overweight and obesity) and accompanying chronic health problems.

The fact child undernutrition in Peru has halved so rapidly implies that the extent or prevalence of the DBM has not (as was feared) risen, but this would be an unwise interpretation; it instead could be a precursor of what is to come if public health and education efforts to curb the processes which lead to adult obesity are not scaled up and made more effective. Social protection programmes in countries in the region, implemented in the 2000s (e.g. *Juntos* in Peru, *Fome Zero* in Brazil) [38, 39] had a shared aim of tackling hunger and food insecurity; the reduction in child malnutrition, and concurrent rise in adult obesity, across all socioeconomic groups and geographic regions (urban/rural) suggests a degree of success but also the possibility of unintended consequences.

### Strengths and limitations

This study used high quality household- and individual-level data from a nationally representative survey, pooling data from multiple rounds collected using standardised methods. Anthropometric measures were taken by well-trained staff ensuring error rates for weight and height measurements were low. The large sample size of the pooled datasets increased power to detect trends and confident inferences regarding the DMB between different sociodemographic groups.

Analyses were all carried out at the child level. This has several advantages compared to household-level analysis. First, nutritional information was available for over 90% of children and their mothers. Second, households sometimes contain multiple families and so allocating a household-level nutritional status to all members (e.g. ‘child underweight and mother overweight’) may be problematic, especially when there are mothers with more than one child in the household. Nutrition status of fathers was not considered in our analysis as data collection on paternal nutrition status started relatively recently thus making assessments of trends part of a future agenda.

Addressing causality will require large-scale longitudinal or implementation studies; the mother–child dyads in our analysis were based on cross-sectional data collected on women aged between 15 and 49, and on their children aged <5 years. Other age groups were not included, and while this could represent a threat to external validity, it in fact increases comparability with other DHS surveys which use the same age criteria. Losses in recruitment, non-response rates and lack of anthropometric measurements were minimal, and appear to be missing at random, so are not expected to affect results. Data on physical activity and detailed diet patterns for child or mothers were not collected.

Although we briefly explored regional variation using the main regions of Peru, future studies should consider more local areas and explore further the amount of individual, household and sub-regional variation in nutritional outcomes. Similarly, a much more in-depth investigation of the impact of education on the risk of overweight and obesity is also needed.

Finally, we note that other problematic aspects of nutritional status, such as anaemia, persist, and are not reflected in our analysis. Since nearly half of Peru’s children under 5 are anaemic there is scope to include it in future work if we are looking for a holistic measure of the double burden [40].

## Conclusions

Across Peru, there has been a substantial decrease in the number of undernourished children, meaning that, on average, the number of DBM cases (i.e. undernourished children with overweight or obese parents) has generally either not grown across the 2000s, or in fact decreased. The risk of having an overweight or obese mother has increased regardless of the child’s nutritional status. We also find little evidence that the composition of this double burden has changed: over 90% of children experiencing the double burden in Peru are stunted, and this has not changed since the mid-1990s. The consistent finding is that children, regardless of their nutritional status, are increasingly more likely to have an overweight mother. Our analyses suggest that exploring the association between maternal nutritional status and the nutritional status of older children (i.e. over the age of 5) should be a focus of future research and policy.

## Supplementary information

Supplementary tables

Supplementary figures

## References

[1] Loret De Mola C, Quispe R, Valle GA, Poterico JA. Nutritional transition in children under five years and women of reproductive age: a 15-years trend analysis in Peru. PLoS One. 2014;9:e92550. 10.1371/journal.pone.0092550 PMC3958518 24643049

[2] Akombi BJ, Chitekwe S, Sahle BW, Renzaho AMN. Estimating the double burden of malnutrition among 595,975 children in 65 low- and middle-income countries: a meta-analysis of demographic and health surveys. Int J Environ Res Public Health. 2019;16:2886. 10.3390/ijerph16162886 PMC6720202 31412530

[3] World Health Organisation. The double burden of malnutrition [Internet]. Geneva: World Health Organisation; 2017 p. 1–10. https://www.who.int/nutrition/publications/doubleburdenmalnutrition-policybrief/en/.

[4] Perez-Escamilla R, Bermudez O, Buccini GS, Kumanyika S, Lutter CK, Monsivais P, et al. Nutrition disparities and the global burden of malnutrition. BMJ [Internet]. 2018;361. https://www.bmj.com/content/361/bmj.k2252. 10.1136/bmj.k2252 PMC5996967 29899012

[5] Shrimpton, R, Rokx C. The double burden of malnutrition: a review of global evidence. Geneva: World Bank; 2012.

[6] Miranda JJ, Barrientos-Gutiérrez T, Corvalan C, Hyder AA, Lazo-Porras M, Oni T, et al. Understanding the rise of cardiometabolic diseases in low- and middle-income countries. Nature Med [Internet]. 2019. 10.1038/s41591-019-0644-7. 10.1038/s41591-019-0644-7 31700182

[7] Kar BR, Rao SL, Chandramouli BA (2008). Cognitive development in children with chronic protein energy malnutrition. Behav Brain Functions.

[8] Peixoto Paes-Silva R, Correia de Macedo EM, Oliveira Tomiya MT, Machado Barbosa de CastroCM (2015). Immune response of severe malnutrition treated according to the World Health Organization. Nutr Hosp.

[9] Sowers JR (2003). Obesity as a cardiovascular risk factor. Am J Med.

[10] Kulkarni VS, Kulkarni VS, Gaiha R (2017). “Double burden of malnutrition”: reexamining the coexistence of undernutrition and overweight among women in India. Int J Health Serv.

[11] Freire WB, Waters WF, Rivas-Mariño G, Belmont P (2018). The double burden of chronic malnutrition and overweight and obesity in Ecuadorian mothers and children, 1986–2012. Nutr Health.

[12] Pomati M, Nandy S. Assessing progress towards SDG2: trends and patterns of multiple malnutrition in young children under 5 in West and Central Africa. Child Indic Res [Internet]. 2019. 10.1007/s12187-019-09671-1.

[13] Templin T, Cravo Oliveira Hashiguchi T, Thomson B, Dieleman J, Bendavid E (2019). The overweight and obesity transition from the wealthy to the poor in low- and middle-income countries: a survey of household data from 103 countries. PLOS Med.

[14] Huayanay-Espinoza CA, Quispe R, Poterico JA, Carrillo-Larco RM, Bazo-Alvarez JC, Miranda JJ (2017). Parity and overweight/obesity in Peruvian women. Prev Chronic Dis.

[15] Loret De Mola C, Quispe R, Valle GA, Poterico JA. Nutritional transition in children under five years and women of reproductive age: a 15-years trend analysis in Peru. PloS One, 9:e92550. 10.1371/journal.pone.0092550 PMC3958518 24643049

[16] Mispireta ML, Rosas ÁM, Velásquez JE, Lescano AG, Lanata CF. Transición nutricional en el Perú, 1991–2005. Rev Peruana Med Exp Salud Publ. 2002;24:129–35.

[17] Dieffenbach S, Stein AD (2012). Stunted child/overweight mother pairs represent a statistical artifact, not a distinct entity. J Nutr.

[18] Instituto Nacional de Estadística e Informática. Peru—Encuesta Demografica y de Salud Familiar [Internet]. 2019. https://proyectos.inei.gob.pe/endes/.

[19] Instituto Nacional de Estadística e Informática. Peru—Encuesta Demografica y de Salud Familiar 1996 [Internet]. Calverton, Maryland, USA: Macro International Inc.; 1997. https://dhsprogram.com/pubs/pdf/FR87/FR87.pdf. Accessed 8 Oct 2020.

[20] Instituto Nacional de Estadística e Informática. Peru—Encuesta Demografica y de Salud Familiar 2000 [Internet]. Lima, Peru: Macro International Inc.; 2001. https://dhsprogram.com/pubs/pdf/FR120/FR120.pdf. Accessed 8 Oct 2020.

[21] Instituto Nacional de Estadística e Informática. Peru—Encuesta Demografica y de Salud Familiar 2004–2006 [Internet]. Lima, Peru: Instituto Nacional de Estadística e Informática; 2007. https://www.dhsprogram.com/pubs/pdf/FR198/FR198.pdf. Accessed 8 Oct 2020.

[22] Instituto Nacional de Estadística e Informática. Peru—Encuesta Demografica y de Salud Familiar 2011 [Internet]. Lima, Peru: Instituto Nacional de Estadística e Informática; 2012. https://proyectos.inei.gob.pe/web/biblioineipub/bancopub/Est/Lib1027/index.html. Accessed 8 Oct 2020.

[23] Instituto Nacional de Estadística e Informática. Peru—Encuesta Demografica y de Salud Familiar 2014 [Internet]. Lima, Peru: Instituto Nacional de Estadística e Informática; 2015. https://www.inei.gob.pe/media/MenuRecursivo/publicaciones_digitales/Est/Lib1211/pdf/Libro.pdf. Accessed 8 Oct 2020.

[24] Kimani-Murage EW, Muthuri SK, Oti SO, Mutua MK, van de Vijver S, Kyobutungi C (2015). Evidence of a double burden of malnutrition in urban poor settings in Nairobi, Kenya. PLoS One.

[25] Kosaka S, Umezaki M (2017). A systematic review of the prevalence and predictors of the double burden of malnutrition within households. Br J Nutr.

[26] Modjadji P, Madiba S. Childhood undernutrition and its predictors in a rural health and demographic surveillance system site in South Africa. Int J Environ Res Public Health. 2019;16. 10.3390/ijerph16173021 PMC6747220 31438531

[27] Hong SA. Prevalence and regional variations of coexistence of child stunting and maternal overweight or obesity in Myanmar. Public Health Nutr. 2020;1–11. 10.1017/S136898002000186X PMC10195513 32677600

[28] De Onis M, Onyango A, Borghi E, Siyam A, Blössner M, Lutter C (2012). Worldwide implementation of the WHO child growth standards. Public Health Nutr.

[29] World Health Organization. WHO child growth standards: length/height-for-age, weight-for-age, weight-for-length, weight-for-height and body mass index-for-age: methods and development. World Health Organization; 2006.

[30] De Onis M, Onyango AW, Borghi E, Garza C, Yang H, WHO Multicentre Growth Reference Study Group. (2006). Comparison of the World Health Organization (WHO) Child Growth Standards and the National Center for Health Statistics/WHO international growth reference: implications for child health programmes. Public Health Nutr.

[31] Fox J, Monette G (1992). Generalized collinearity diagnostics. J Am Stat Assoc.

[32] Fakier A, Petro G, Fawcus S (2017). Mid-upper arm circumference: a surrogate for body mass index in pregnant women. South Afr Med J.

[33] Pries AM, Rehman AM, Filteau S, Sharma N, Upadhyay A, Ferguson EL (2019). Unhealthy snack food and beverage consumption is associated with lower dietary adequacy and length-for-age z-scores among 12–23-month-olds in Kathmandu Valley, Nepal. J Nutr.

[34] Kraak VI, Story M (2015). Influence of food companies’ brand mascots and entertainment companies’ cartoon media characters on children’s diet and health: a systematic review and research needs. Obes Rev.

[35] Gea-Horta T, Silva R, de CR, Fiaccone RL, Barreto ML, Velasquez-Melendez G (2016). Factors associated with nutritional outcomes in the mother-child dyad: a population-based cross-sectional study. Public Health Nutr.

[36] World Health Organization. Double burden of malnutrition (2017). https://www.who.int/nutrition/double-burden-malnutrition/en/. Accessed 23 Dec 2019.

[37] Zhou B, Lu Y, Hajifathalian K, Bentham J, Di Cesare M, Danaei G (2016). Worldwide trends in diabetes since 1980: a pooled analysis of 751 population-based studies with 4·4 million participants. The Lancet.

[38] Carrillo-Larco RM, Miranda JJ, Bernabé-Ortiz A (2016). Impact of food assistance programs on obesity in mothers and children: a prospective cohort study in Peru. Am J Public Health.

[39] Pérez‐Lu JE, Cárcamo C, Nandi A, Kaufman JS (2017). Health effects of ‘Juntos’, a conditional cash transfer programme in Peru. Matern Child Nutr.

[40] Accinelli RA, Leon-Abarca JA (2020). Age and altitude of residence determine anemia prevalence in Peruvian 6 to 35 months old children. PloS One.

