# Supplementary material for: Trends and patterns of the double burden of malnutrition (DBM) in Peru: a pooled analysis of 129,159 mother–child dyads
Source: Int J Obes (Lond). 2021 Jan 5;45(3):609–18. doi: 10.1038/s41366-020-00725-x (PMC7906898; doi:10.1038/s41366-020-00725-x)
Supplement: Supplementary file 1 — Supplementary tables [file 41366_2020_725_MOESM1_ESM.docx]

**Online Supplement**

**TABLE A1:** Logistic regression model for probability of having overweight or obese mother (2015–2017), Odds ratios.

|  | **Model 1** | **Model 2** | **Model 3** | **Model 4** |
| --- | --- | --- | --- | --- |
| (Intercept) | 1.74 (0.05)^***^ | 1.85 (0.08)^***^ | 1.86 (0.08)^***^ | 1.86 (0.09)^***^ |
| Age (30 months) | 1.01 (0.00)^***^ | 1.00 (0.00) | 1.00 (0.00) | 1.00 (0.00) |
| Year (2015) |  |  |  |  |
| 2016 | 0.99 (0.03) | 1.00 (0.03) | 1.00 (0.03) | 1.00 (0.03) |
| 2017 | 1.03 (0.03) | 1.05 (0.03) | 1.05 (0.03) | 1.05 (0.03) |
| Child Nutritional Status (Normal) |  |  |  |  |
| Overweight/Obese | 1.76 (0.09)^***^ | 1.81 (0.09)^***^ | 1.81 (0.09)^***^ | 1.80 (0.14)^***^ |
| Undernourished | 0.74 (0.02)^***^ | 0.71 (0.02)^***^ | 0.71 (0.02)^***^ | 0.82 (0.09) |
| Rural | 0.64 (0.02)^***^ | 0.63 (0.02)^***^ | 1.32 (0.24) | 1.12 (0.21) |
| Wealth Index (Rich) |  |  |  |  |
| Middle | 1.11 (0.04)^**^ | 1.13 (0.04)^**^ | 1.11 (0.04)^**^ | 1.12 (0.05)^**^ |
| Poor | 1.02 (0.03) | 1.08 (0.04) | 1.12 (0.04)^**^ | 1.13 (0.05)^**^ |
| Mother's age (30) |  | 1.08 (0.00)^***^ | 1.08 (0.00)^***^ | 1.08 (0.00)^***^ |
| Mother’s highest level of education (Primary) |  |  |  |  |
| No education |  | 0.63 (0.06)^***^ | 0.64 (0.06)^***^ | 0.64 (0.06)^***^ |
| Secondary |  | 1.00 (0.03) | 0.99 (0.03) | 0.99 (0.03) |
| Higher |  | 0.62 (0.03)^***^ | 0.61 (0.03)^***^ | 0.61 (0.03)^***^ |
| *Interaction Effects* |  |  |  |  |
| Overweight/Obese: Rural |  |  | 0.76 (0.16) | 0.89 (0.20) |
| Undernourished: Rural |  |  | 0.45 (0.08)^***^ | 0.54 (0.10)^**^ |
| Overweight/Obese:Middle |  |  |  | 8.46 (7.01)^*^ |
| Undernourished:Middle |  |  |  | 2.27 (1.60) |
| Overweight/Obese:Poor |  |  |  | 0.93 (0.13) |
| Undernourished:Poor |  |  |  | 0.95 (0.13) |
| Rural: Middle |  |  |  | 1.06 (0.14) |
| Rural:Poor |  |  |  | 0.85 (0.10) |
| Overweight/Obese:Rural:Middle |  |  |  | 0.13 (0.12)^*^ |
| Undernourished:Rural:Middle |  |  |  | 0.40 (0.31) |
| Overweight/Obese:Rural:Poor |  |  |  | 0.12 (0.10)^*^ |
| Undernourished:Rural:Poor |  |  |  | 0.42 (0.30) |
| Num. obs. | 65593 | 65551 | 65551 | 65551 |
| AIC | 85918 | 82162 | 82104 | 82114 |
| Adj.Rsq (Nagelkerke) | 0.03 | 0.11 | 0.11 | 0.11 |
| ***p < 0.001, **p < 0.01, *p < 0.05 Standard errors in brackets. Reference Categories in Brackets | | | | |
