# Supplementary material for: Trends and patterns of the double burden of malnutrition (DBM) in Peru: a pooled analysis of 129,159 mother–child dyads
Source: Int J Obes (Lond). 2021 Jan 5;45(3):609–18. doi: 10.1038/s41366-020-00725-x (PMC7906898; doi:10.1038/s41366-020-00725-x)

**FIGURE A1 Percentage of children according to child and mother nutritional status by region (1995-2010)**


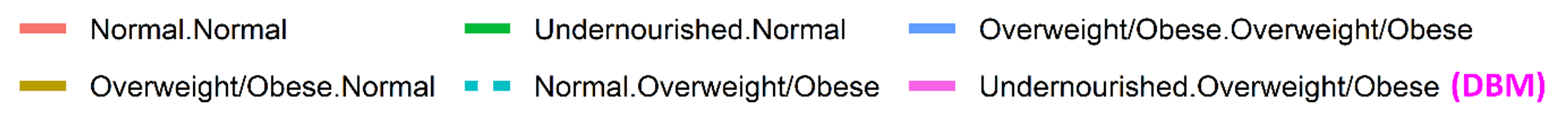


The blue dashed line shows the percentage of normal children with overweight or obese mothers, whereas the purple lines shows the percentage of double burden of malnutrition (DBM) children (i.e. undernourished children with overweight or obese mothers).


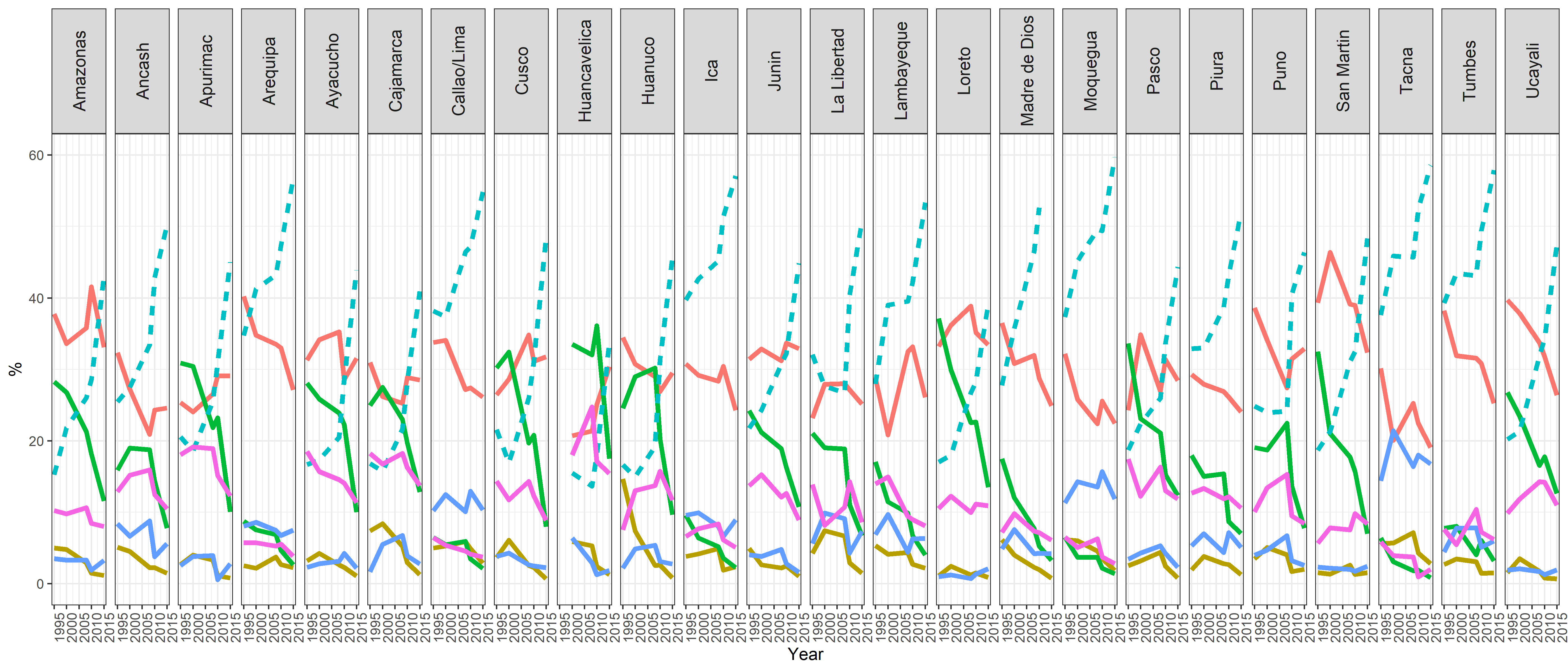


**FIGURE A2 Percentage of children according to child and mother nutritional status by rural/urban and wealth index status (1995-2010)**


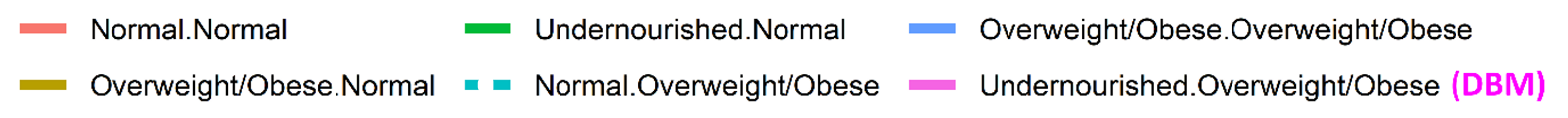


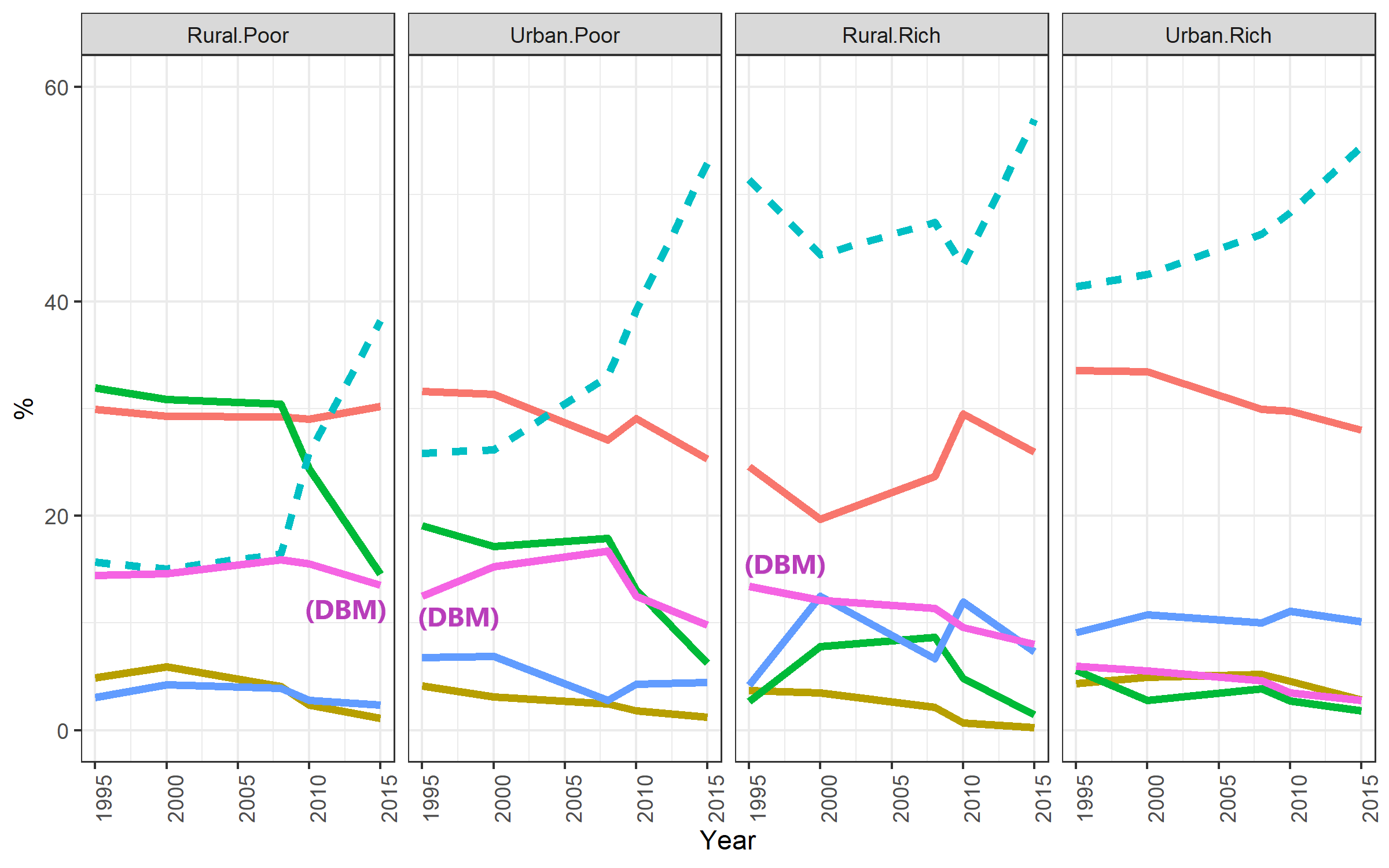


The blue dashed line shows the percentage of normal children with overweight or obese mothers, whereas the purple lines shows the percentage of double burden of malnutrition (DBM) children (i.e. undernourished children with overweight or obese mothers).

**Figure A3 Percentage of children according to child and mother nutritional status by level of maternal education and wealth index status (1995-2010)**


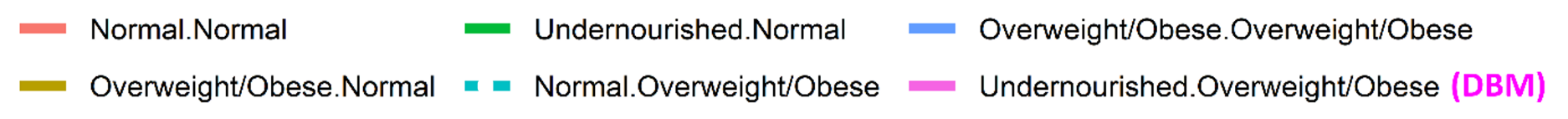


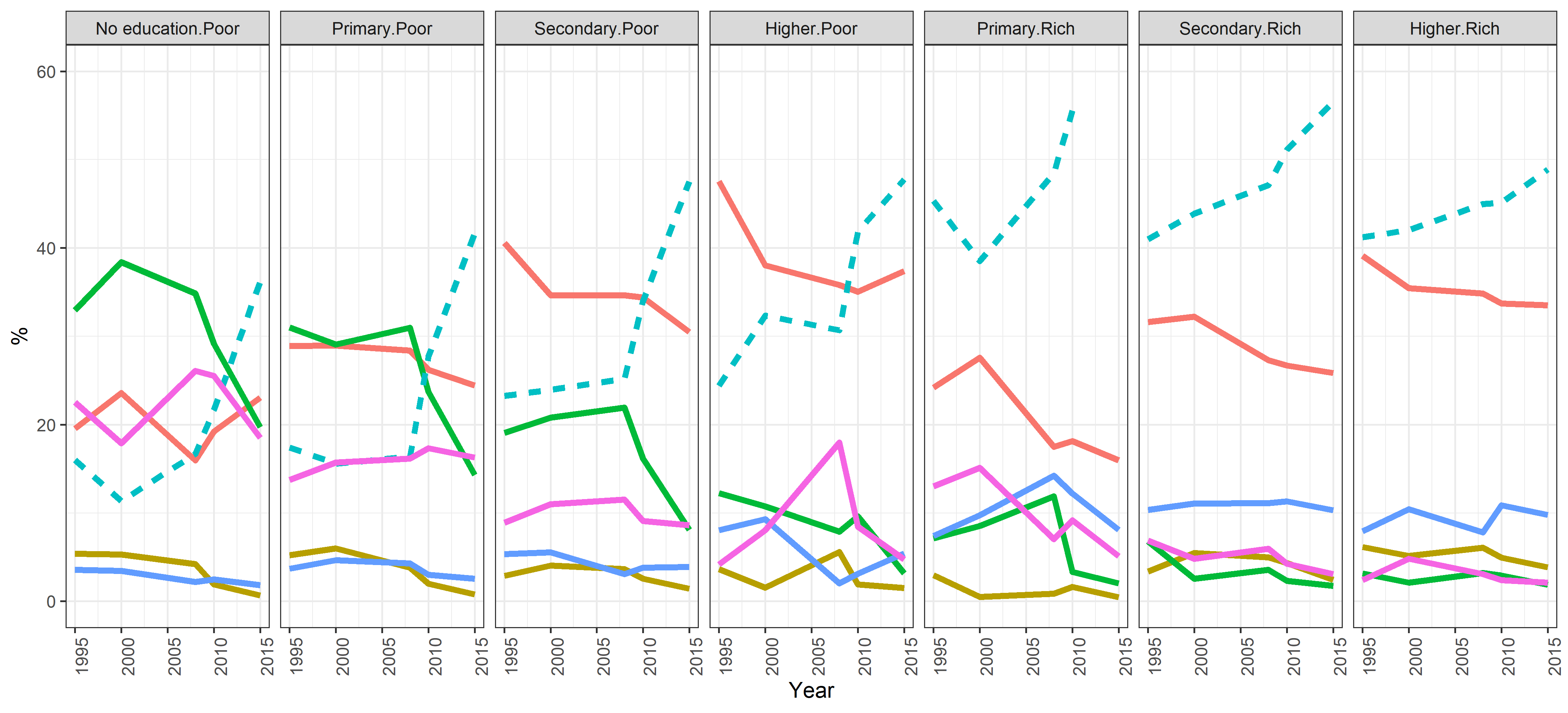


The blue dashed line shows the percentage of normal children with overweight or obese mothers, whereas the purple lines shows the percentage of double burden of malnutrition (DBM) children (i.e. undernourished children with overweight or obese mothers).

**FIGURE A4 Predicted probability of having an overweight or obese mother by child’s nutritional status, rural/urban and wealth index status from logistic regression model (Table A1, Model 4)**


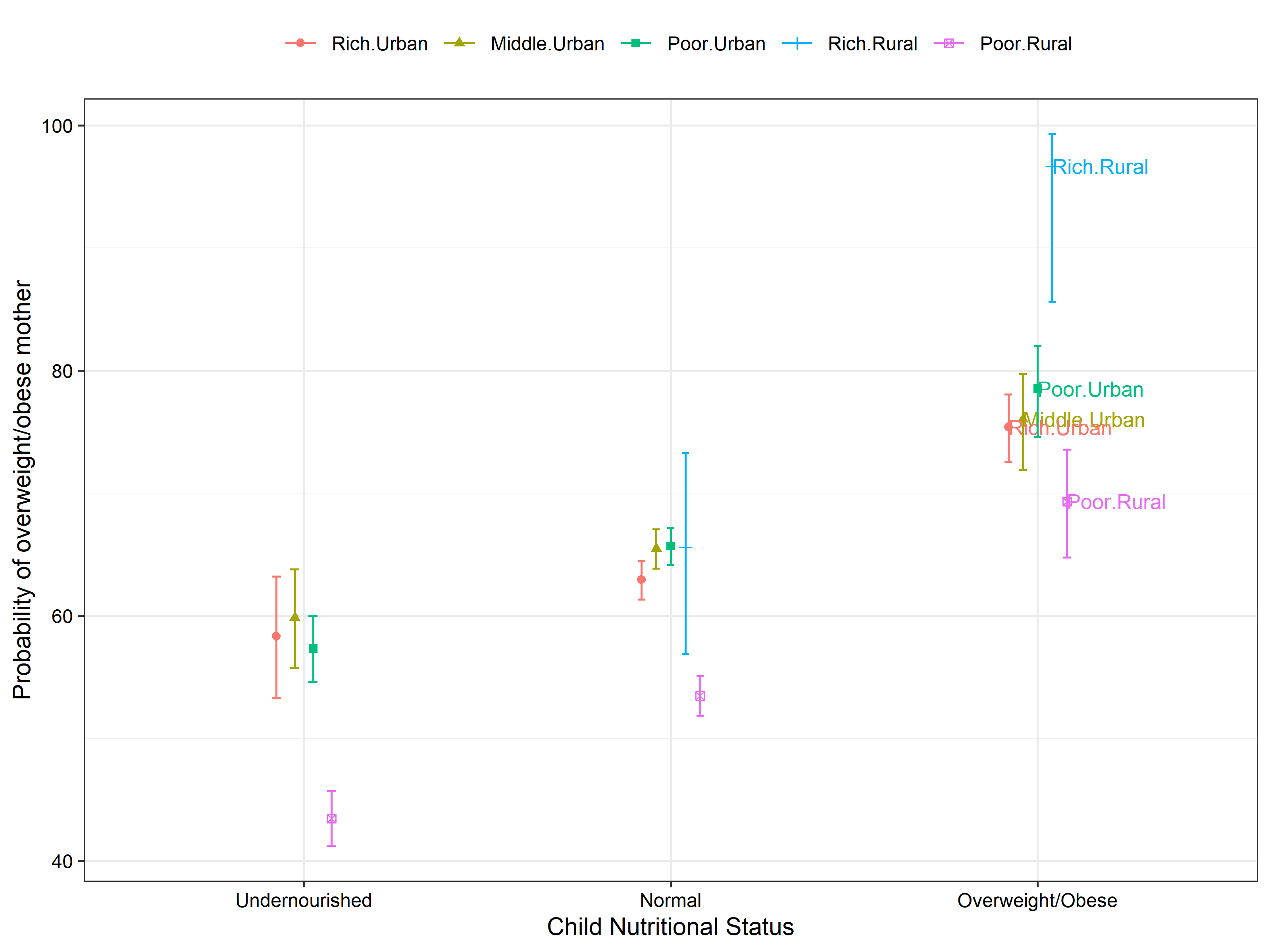

Supplement: Supplementary file 2 — Supplementary figures [file 41366_2020_725_MOESM2_ESM.docx]
